# A 16‐year‐old female with an intrasellar mass

**DOI:** 10.1111/bpa.70002

**Published:** 2025-03-04

**Authors:** Yinbo Xiao, Can Yin, Junliang Lu, Shuangni Yu, Zhen Huo, Zhiyong Liang

**Affiliations:** ^1^ Department of Pathology Molecular Pathology Research Center, Peking Union Medical College Hospital, Chinese Academy of Medical Science & Peking Union Medical College Beijing China; ^2^ Department of Pathology Hebei General Hospital Shijiazhuang China

**Keywords:** DICER1, DICER1 mutant, pituitary neuroendocrine tumor/pituitary adenoma, primary intracranial sarcoma

BOX 1 **Virtual glass slide**

Access at https://isn‐slidearchive.org/?col=ISN&fol=Archive&file=BPA‐24‐07‐CI‐180‐3.svs and https://isn‐slidearchive.org/?col=ISN&fol=Archive&file=BPA‐24‐07‐CI‐180‐R4.svs.

## 1 CLINICAL HISTORY AND IMAGING

**FIGURE 1:**
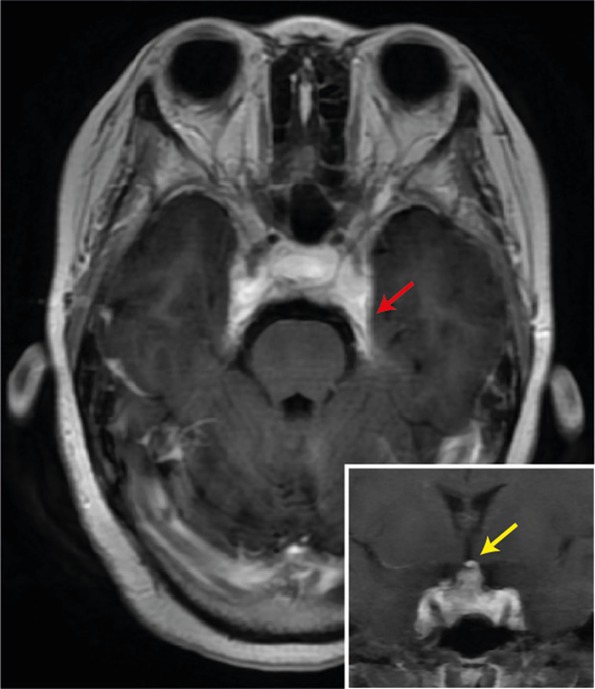
Imaging feature. T1‐weighted contrast‐enhanced MRI showed a heterogeneous enhancing sellar mass. This lesion invaded the bilateral cavernous sinuses (red arrow, axial view) and protruded into the suprasellar cistern (yellow arrow, coronal view, inset).

A 16‐year‐old girl presented to the clinic with a 2‐year history of weight gain. In the last week, she had developed blepharoptosis and blurred vision, with headache and vomiting. She did not have a prior history of tumors. Physical examination showed a moon face, buffalo hump, and purpura. Laboratory findings showed elevated 24‐hour urinary free cortisol (UFC) and plasma adrenocorticotropic hormone (ACTH). The low‐dose dexamethasone suppression test failed to suppress cortisol production, but the high‐dose test did. Magnetic resonance imaging (MRI) revealed a 15.9 × 10.1 × 12.8 mm^3^ sellar mass with heterogeneous T1 signal enhancement (Figure 1). It extended into the bilateral cavernous sinuses and the suprasellar cistern, impacting the optic chiasm. Surgery was pursued for a definitive diagnosis and tumor debulking (Box 1).

## 2 FINDINGS

**FIGURE 2:**
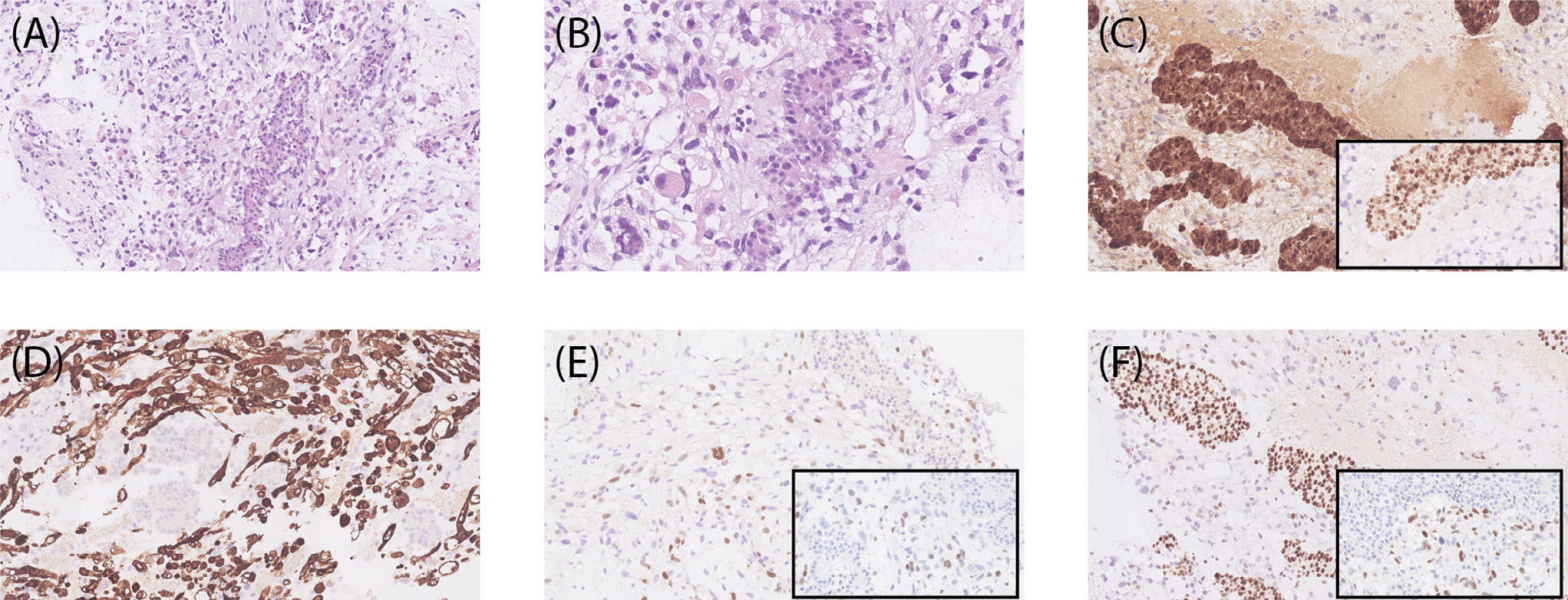
The histological features of the lesion. Hematoxylin and eosin (H&E) staining showed the lesion containing two neoplasm components (A, ×200; B, ×400). The epithelial cells were small and uniform with solid or glandular structures, which were positive for ACTH (C, ×200), T‐PIT (C, inset, ×200), ATRX (F, ×200), but negative for P53 (F, inset, ×200). Mesenchymal cells were spindle‐shaped with eccentric nuclei, containing prominent eosinophilic cytoplasmic globules (B), which were positive for Desmin (D, ×200), Myogenin (E, ×200), MyoD1 (E, inset, ×200), P53 (F, inset, ×200), but negative for ATRX (F, ×200).

Histopathological examination revealed a biphasic tumor with a neuroendocrine and a mesenchymal component. The neuroendocrine component, with well‐differentiated solid or glandular structures, showed positive staining for Synaptophysin (Syn), ACTH, and transcription factors T‐PIT and INSM‐1 but negative for PIT‐1 and SF‐1 (Figure 2). The mesenchymal component in the stromal background was composed of spindle or irregular cells with large nuclei and abundant eosinophilic cytoplasm. These tumor cells were positive for Desmin, MyoD1, and Myogenin (Figure 2). Unlike the neuroendocrine component, the mesenchymal component exhibited strong nuclear p53 positivity and loss of ATRX expression. Mitoses were frequent in the mesenchymal component (up to 8 per 10 HPF). The mitotic count was in keeping with the Ki67 labeling (the Ki67 index was significantly higher in the mesenchymal components [50%] compared with the epithelial cells [3%]). NGS testing identified several pathological mutations as follows: *DICER1* (c.4860dup, p.Cys1621Leufs*31), *DICER1* (c.5428G>T, p.Asp1810Tyr), *TP53* (c.740A>T, p.N247I), *ATRX* (c.594+1G>T), and *PIK3CA* copy‐number gain.

## 3 DIAGNOSIS

Corticotrophin tumor/adenoma in association with primary intracranial sarcoma, DICER1‐mutant.

## 4 DISCUSSION

This is a unique case of a young patient who presented with signs and symptoms of Cushing's disease and was diagnosed post‐surgery as “corticotrophin tumor/adenoma associated with primary intracranial sarcoma, DICER1‐mutant.” Primary intracranial sarcoma, DICER1‐mutant (PIS‐DICER1) is a rare molecularly defined entity. According to the 2021 World Health Organization (WHO) CNS tumor classification, it is defined as a primary intracranial sarcoma with distinctive morphology, typically showing immunophenotypic myogenic differentiation [1]. DICER1 mutations are definite features, commonly along with TP53 mutations and ATRX inactivation. These tumors usually occur in young patients (median age at diagnosis as 6 years) and appear to be aggressive, notably at risk for DICER1 syndrome. Without appropriate genetic testing, these neoplasms may be easily misclassified as “rhabdomyosarcoma,” potentially underestimating their clinical implications.

The present tumor was initially suspected to be a corticotrophin tumor/adenoma based on Cushing's manifestations and histological findings, which showed well‐differentiated nests of pituitary cells with positive Syn, INSM‐1, and T‐PIT lineage markers (including T‐PIT and ACTH). However, the discovery of rhabdomyoblast‐like cells with positive skeletal muscle differentiation markers (Desmin, MyoD1, and Myogenin) in the stromal background, raised the possibility of alternative diagnoses, such as PIS‐DICER1 or rhabdomyosarcoma. Consequently, the final diagnosis of “corticotroph tumor/adenoma associated with primary intracranial sarcoma, DICER1‐mutant” was made after confirming the presence of DICER1 mutations. Such a case is exceedingly rare. To date, only four cases of sellar rhabdomyosarcoma in association with pituitary adenomas have been previously reported [2, 3]. All these were in adults, ranging in age from 34 to 77 years, and one case had a history of prior radiation exposure. Unlike these cases, PIS‐DICER1 is more common in younger patients. For this age group and DICER1 mutation, pituitary blastoma (PitB) should be included as a differential diagnosis. However, morphologic features of PitB, such as undifferentiated blastemal cells and Rathke's pouch epithelium are not seen in this case, thereby not being considered. Another differential diagnosis, DICER1‐associated rhabdomyosarcoma, was excluded since no cases of intracranial DICER1‐associated rhabdomyosarcoma have been reported.

The histogenesis of PIS‐DICER1 and its association with corticotrophin tumor/adenoma remain unclear. Further investigation is required to separately assess the mutation profiles of both tumor components, which may provide additional insights into the pathogenesis of such complex tumors.

Our patient received chemotherapy after surgery and was kept alive with no evidence of disease after 8 months. Currently, there is no indication of DICER1 tumor predisposition syndrome in this case; however, continued monitoring and follow‐up are warranted.

## AUTHOR CONTRIBUTIONS

Yinbo Xiao wrote the main manuscript text. Can Yin prepared the figures. Junliang Lu performed NGS testing. Shuangni Yu, Zhen Huo, and Zhiyong Liang reviewed and edited the manuscript. All authors approved the final version of the paper.

## FUNDING INFORMATION

This work was supported by the National High‐Level Hospital Clinical Research Funding (grant number 2022‐PUMCH‐B‐063).

## CONFLICT OF INTEREST STATEMENT

The authors declare that there is no conflict of interest.

## ETHICS STATEMENT

The studies were approved by the Ethics Review Committee of the Peking Union Medical College Hospital (Ethics Certificate No. K2750).

## Data Availability

The data that support the findings of this study are available from the corresponding author upon reasonable request.
